# Clinical Outcome After Surgical Treatment of Traumatic Peroneal Nerve Injury: An Analysis of Risk Factors After Different Surgical Approaches

**DOI:** 10.3390/neurolint17010007

**Published:** 2025-01-13

**Authors:** Daniel N. Werkmann, Ute M. Bäzner, Martin Petkov, Lena Minzenmay, Gregor Durner, Gregor Antoniadis, Christian R. Wirtz, Maria T. Pedro, Andreas Knoll, Andrej Pala

**Affiliations:** 1 Department of Neurosurgery, University of Ulm, BKH Günzburg, Lindenallee 2, 89312 Günzburg, Germany; daniel.werkmann@uni-ulm.de (D.N.W.); lena.federle@uni-ulm.de (L.M.); gregor.durner@googlemail.com (G.D.); rainer.wirtz@bkh-guenzburg.de (C.R.W.);; 2 Peripheral Nerve Unit, Department of Neurosurgery, BKH Günzburg at Ulm University, Lindenallee 2, 89312 Günzburg, Germany; gregor.antoniadis@uni-ulm.de (G.A.); maria-teresa.pedro@uni-ulm.de (M.T.P.)

**Keywords:** traumatic nerve lesion, peroneal nerve, nerve graft, peripheral nerve

## Abstract

Background: This study aims to analyze potential risk factors that may influence the clinical outcomes following surgical treatment of traumatic peroneal nerve lesions. Methods: We conducted a retrospective analysis of patients with traumatic peroneal nerve injuries treated with decompression, split repair, or nerve grafting between 2010 and 2020. Motor function and potential risk factors were evaluated. Results: Out of 93 patients, 42 (45%) underwent decompression, 15 (16%) received split repair, and 36 (39%) required autologous nerve grafting. Up to one year after surgery, weakness of the anterior tibial muscle improved from a median of M0 to M3. After one year following nerve decompression, functional recovery was observed in 28 (65%) cases, in 9 (21%) cases after split repair, and in 7 (16%) cases following autologous nerve grafting. A defect greater than 8 cm was associated with significantly poorer improvement of extensor hallucis longus (*p* = 0.037, HR 0.109). We found no significant associations between age, diabetes mellitus, arterial hypertension, obesity, and postoperative outcomes. Conclusions: According to the present data, a significant number of patients achieved functional improvement following surgical treatment, indicating that this procedure should be considered an important treatment option in selected cases.

## 1. Introduction

Traumatic or iatrogenic lesions of peripheral nerves are a rather common pathology. Although the nerves of the lower extremity are less frequently affected, the peroneal nerve is most frequently afflicted resulting in foot drop [1]. The regenerative ability of the peroneal nerve due to its anatomical features and vascularization is often related to inferior outcomes [2,3]. Despite the low incidence of peroneal nerve lesions, a persistent loss of function can result in immobility, pain, and lower quality of life with additional socioeconomic impacts [4,5].

The goal of this study is to analyze the early functional outcome of patients after surgical treatment of traumatic peroneal nerve injury as well as to evaluate the effect of possible risk factors associated with worse clinical outcomes, such as different states of obesity or cardio-vascular risk factors like smoking, elevated blood pressure, and diabetes. Factors relating to the pathogenesis and surgical treatment were also evaluated. The intention of gathering these data is to improve the decision-making process regarding which patients to administer nerve reconstruction surgery and in which cases a conservative or primarily plastic surgical approach is expedient.

## 2. Materials and Methods

We performed a retrospective, monocenter study including all patients who underwent surgical treatment after peroneal nerve injury between 2010 and 2020 at our department. The study was approved by the Ethical Review Committee at the University of Ulm, Germany (400/21).

### 2.1. Patient Data

Clinical data were reviewed retrospectively. General characteristics and trauma genesis were noted. A total of 93 patients were examined clinically pre- and post-surgery. After surgery, the follow-up intervals were set up to 6 months, 1 year, and more than 1 year after surgery. To determine the motor function deficit, the grading system of the Medical Research Council (MRC) was used, dividing the muscle strength into grades from 0 to 5 [6]. All muscles innervated by the peroneal nerve were examined, with the extensor hallucis longus muscle (EHL), extensor digitorum communis muscle (EDC), peroneus longus muscle (PM), and anterior tibial muscle (ATM) serving as the main muscles to determine a foot-drop syndrome and hereby functional limitation. The main point of interest was the difference in the post-operative MRC grades in relation to the pre-operative grading. Additionally, sensory deficits, pain dichotomized as present or not present, were gathered. The type of surgery and length of the nerve lesion according to the findings in intraoperative ultrasound were evaluated. In the case of nerve reconstruction, the length and the donor nerves, as well as the number of nerve grafts, were evaluated. Furthermore, the body mass index, arterial hypertension, age, diabetes mellitus, history of smoking, and sex were considered as potential risk factors.

As a part of the standard procedure, all patients were counseled to undergo physiotherapy at least 3 times per week for 30 min or longer before surgery and after surgery. In the case of nerve reconstruction with grafting, physiotherapy is recommended no earlier than two weeks after the surgery. Unfortunately, due to the retrospective characteristics of the study, it was not possible to evaluate how many patients followed this recommendation.

### 2.2. Surgical Techniques

In our clinic, a center for peripheral nerve surgery, we use 3 main types of reconstructive surgery, similar to the techniques used by Kim and Kline [7]. In mild cases and intact nerve structures, neurolysis is performed, wherein the nerve is dissected and released out of the scar tissue. If parts of the nerve or fascicles are severely damaged, a split repair is necessary. The epineurium is opened and the injured nerve tissue or neuroma is excised and substituted with an autologous nerve. Nerve stimulation is employed during the procedure to identify the functional fascicles. The sural nerve is the most commonly used graft. In severe cases with an extensive nerve injury or a loss of continuity, an autologous nerve graft typically from the sural nerve is used. One or multiple nerve grafts were used as interpositions for the injured section fixed via epineural reconstruction with 10/0 sutures and fibrin glue.

### 2.3. Statistical Analysis

Statistical analysis was performed using SPSS 29.0 (Lead Technologies, Inc., Charlotte, NC, USA). Descriptive statistics as well as univariable logistic regression were used. Because of the low number of patients and the fact that only one variable achieved a significant difference in univariable analysis, multivariable analysis was excluded. The significance level was set as *p* < 0.05.

## 3. Results

Of the 93 patients included in the study, all of them were treated by surgery, divided into 42 patients (45%) receiving neurolysis, 15 (16%) receiving split-repair, and 36 (39%) undergoing autologous nerve grafting, as shown in Table 1.

Before surgery, 84 patients (90%) presented with severe paresis (M ≤ 2) and 67 (72%) with plegia (M = 0). Moreover, 88 (95%) patients reported sensory deficits and 20 (22%) were experiencing pain.

**Figure 1:**
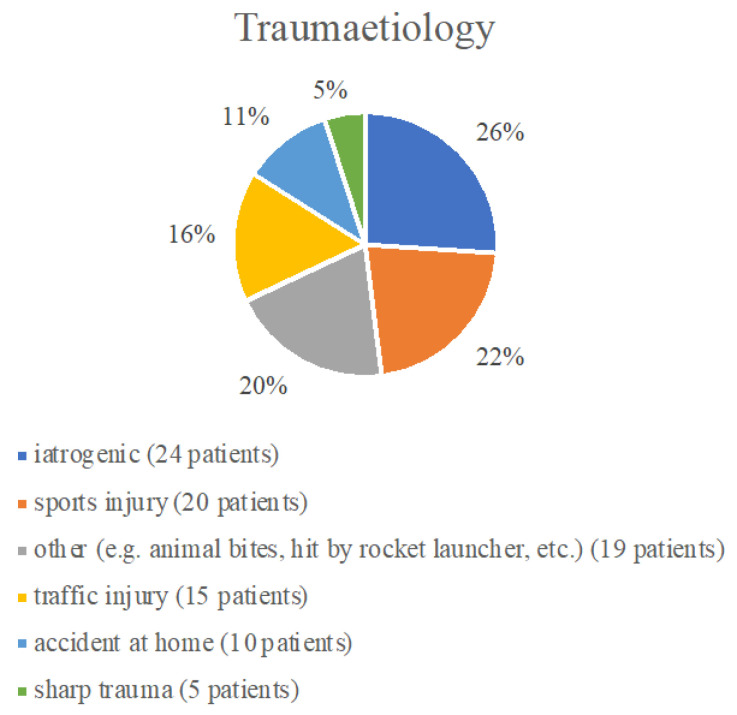
Traum etiology.

The genesis of the evaluated lesions is shown in Figure 1. In 19 (20%) cases, the trauma was not divisible into any of the groups and included etiologies such as animal bites or a rocket launcher.

The median time interval from accident to surgery was 4 months (ranging from 0 to 42), while the median age at the time of surgery was 34 years (ranging from 7 to 78). Table 2 depicts the MRC grades before surgery.

At the time point of more than 1 year after surgery, 44 (47%) patients recovered functionally (ATM, M ≥ 3), while the median of the ATM was 4 (range 0 to 5). Moreover, 31 (33%) patients recovered by M = 3, 9 (10%) by M = 2, and 10 (11%) by M = 1. Lastly, 14 (15%) patients had to use a gait aid (peroneal splint) permanently.

The results of recovery divided into subgroups by type of surgery are shown in Table 3 and Table 4. Table 3 depicts the recovery and Table 4 presents the absolute grades of muscle strength.

More than 1 year after surgery, out of the 88 patients that reported sensory deficits, 26 (30%) improved, while pain relief was achieved in 8 (40%) out of 20 cases.

Regarding risk factors, we found no significant association between the postoperative outcome and the presumptive risk factors of patient age, BMI, smoking, arterial hypertension, and diabetes, as shown in Table 5, Table 6 and Table 7.

In particular, different stages of obesity could not be correlated significantly to the post-surgical outcome, as shown in Table 8.

In univariable analysis of the risk factors, a graft length of > 8 cm was related to significantly worse outcomes for EHL (*p* = 0.037, HR = 0.109, CI = 0.14–0.872). The time from trauma to surgical treatment showed a tendency toward a worse outcome for the EHL after neurolysis (*p* = 0.064, HR = 0.656, CI = 0.421–1.024).

Table 9 depicts the results of pain and sensory deficits before and after surgery.

## 4. Discussion

The peroneal nerve is the most commonly injured nerve of the lower extremities [1]. The symptoms can cause substantial personal and socioeconomic damage, making surgical reconstruction a valuable treatment option. In several studies, including large patient collectives, key points influencing the outcome were reported [7,8,9,10]. Those studies focused on the influence of the timing of surgery, severity of the lesion, etiology of the trauma, and graft length (Kim et al.) or on socioeconomic background and cardiovascular risk factors (Liu et al.). Unified treatment guidelines could not be developed due to the heterogeneity of the lesions, the patient collectives, and the surgical approaches [11]. When comparing our data to the current literature, we found similar results. Patients with less severe damage showed better recovery. A longer period between trauma and surgery was suggested to have an influence on worse outcomes. In general, we found a tendency toward statistical significance for the time span from trauma to operation relating to recovery of the EHL. Even if it is highly likely that there is a relevance to this factor, as national guidelines recommend a preferably short time to operation, it is only partially reflected in the present study situation, as many studies do not report results regarding this factor. Terzis et al. showed a significantly worse outcome after more than 13 months from trauma to surgery.

According to our results, we found significantly poorer functional outcomes of the EHL with a nerve graft length exceeding 8 cm. These data are similar to our previously published results showing worse outcomes with a graft length above 6 cm [12]. Those graft lengths seem significantly shorter than the ones published by Kim et al. (2004) showing worse outcomes at a graft length above 12 cm, Terzis et al. (2019) showing above 13 cm, and Strother et al. (2023) even when paired with posterior tendon transfer [7,8,12,13]. The retrospective observation interval of our patient collective was set significantly shorter than in the cited studies, with a median of 16 months compared to 30 months (Kim et al.) and 32 months (Terzis et al.). Compared to large meta-analyses, our data seems to fit within the range of 9 cm identified by Mackay et al. (2022) to 12 cm identified by George et al. (2014) [14,15]. The operative techniques used were comparable to Kim et al. In other studies (Terzis et al.), there were partially combined neurosurgical and plastic surgical approaches, which reduces the comparability of the results. Currently, there are no general recommendations at which point a neurosurgical approach should be accompanied by a plastic surgical technique, such as a posterior tibial tendon transfer. Our data postulate that if the nerve lesion exceeds 8 cm in length, a tendon transfer might be a reasonable option.

Concerning the risk factors, we examined general risk factors like age and time to operation, and specifically cardiovascular risk factors, due to the consideration that the peroneal nerve might have a reduced regenerative ability compared to other peripheral nerves due to its vascular supply [2,15]. Additionally, we examined obesity as we expected it to have a negative influence on the outcomes of surgical procedures.

Age and sex showed no statistical significance. For arterial hypertension, diabetes mellitus, and smoking, there was no negative correlation in terms of the functional outcome. Peripheral artery disease did not occur in our patient collective. Liu et al. showed a significant effect of diabetes mellitus type 2 on worse outcomes for a large Chinese collective [9]. There is likely no final assessment possible for the relevance of diabetes as a risk factor, citing this study or our present data, because of the retrospective design of both studies and the low overall expression of diabetes in the patient collectives (16% in our collective and 3% in Liu et al.’s patients). Additionally, it may be possible that the diseases caused by the investigated vascular risk factors did not have an effect due to the low median age of 34 years of the patients included in our study.

Upon investigating different stages of obesity, there was also no negative effect shown on outcomes. No higher rate of peri- or postoperative complications was shown either. This may be caused by the obesity paradox, first described in gastroenterological cancer surgery, showing an advantage for patients with higher BMI against postoperative mortality, and in some cases, morbidity, compared to patients with lower BMI, although this is not the case for all general surgeries [16,17]. Another possibility is the perineural fat tissue serving as a fat envelope, creating favorable conditions for the nerve, similar to the ulnar nerve as described by Riccio et al. [18].

### Limitations of This Study

This was a single-center retrospective study over the course of 10 years, with the acquisition of data from physical and electronic patient records. There were different surgeons working in our department taking the anamnesis and performing the physical examination of the patients, creating a heterogenic data situation, with some MRC grades or other information missing in some files. An even more heterogenic data situation existed for the electrophysiological and imaging diagnostics if present, as the patients brought the results along in most cases and the examinations were, in part, performed by colleagues not acquainted with our department.

As a well-known center of peripheral nerve surgery, we treated patients over the whole federal territory so that the patients did not keep their set appointments at our department at all times, especially in cases with very good or very bad outcomes and a long distance to travel for the patients. Finally, we included the most current data acquired after surgery in a median time span of 16 months, understanding that the patients treated by nerve graft especially may still recover and could not be evaluated at this final time point.

## 5. Conclusions

Surgical treatment of peroneal nerve injuries may lead to good functional improvements, reduced pain, and improved sensory deficits. According to our data, only the length of the nerve graft was identified as a potential risk factor. Even if the surgical methods used today can already provide a significant recovery for the patients, further studies are needed to evaluate risk factors influencing the outcomes.

## Figures and Tables

**Table 1:** Patient characteristics.

	**No.**	**Percent**	**Median**	**Min**	**Max**
Patients					
Male	63	68			
Female	30	32			
Age (years)			34	7	78
Left side injury	49	53			
Right side injury	44	47			
Risk factors					
Arterial hypertension	15	16			
Diabetes mellitus	9	10			
Smoking	16	17			
BMI (kg/m^2^)			25.26	14.31	45.63
Surgical factors					
Time: Trauma-surgery (month)			4	0	42
Trauma surgery prior	75	80			
Neurolysis	42	45			
Split-Repair	15	16			
Autologous Nerve graft	36	39			

**Table 2:** Strength of the muscles in MRC grades before surgery.

	ATM						EHL						EDC						PM					
MRC	0	1	2	3	4	5	0	1	2	3	4	5	0	1	2	3	4	5	0	1	2	3	4	5
Neurolysis	26	9	2	3	0	2	23	7	5	2	1	2	15	7	4	3	0	0	18	3	4	4	6	2
Split-Repair	7	2	2	0	1	2	10	0	2	1	0	2	6	2	1	0	0	0	6	1	1	2	0	3
Nerve graft	34	2	0	0	0	0	34	2	0	0	0	0	28	0	0	0	0	0	29	2	1	1	2	0

**Table 3:** Development of muscle strength more than 1 year after surgery.

	ATM			EHL		EDC		PM	
	M + 1	M + 2	M + 3	M + 1	M + 2	M + 1	M + 2	M + 1	M + 2
Neurolysis (42)	0	5	20	3	18	4	10	8	15
Split-Repair (15)	2	2	4	4	4	1	3	0	8
Nerve graft (36)	8	1	7	3	6	3	4	4	8

**Table 4:** Strength of the muscles in MRC grades more than 1 year after surgery.

	ATM						EHL						EDC						PM					
MRC	0	1	2	3	4	5	0	1	2	3	4	5	0	1	2	3	4	5	0	1	2	3	4	5
Neurolysis	5	1	1	6	12	10	6	6	2	7	10	3	5	2	3	7	5	2	5	0	2	4	6	17
Split-Repair	2	0	1	3	3	3	1	4	2	3	1	1	1	2	2	4	1	0	0	0	0	5	2	4
Nerve graft	19	8	2	2	2	3	25	2	3	4	0	0	18	4	1	3	1	0	21	2	3	2	4	2

**Table 5:** Effect of risk factors on outcome of neurolysis showing the significance level *p*, the hazard ratio HR, and the confidence interval CI.

Neurolysis		ATM			EHL			PM	
	*p*	HR	CI	*p*	HR	CI	*p*	HR	CI
Obesity	0.584	0.960	0.828–1.112	0.468	0.955	0.842–1.082	0.211	1.092	0.951–1.252
Arterial Hypertension	0.827	0.762	0.067–8.726	0.227	0.292	0.040–2.150	0.999	-	-
Diabetes mellitus	1	-	-	1	-	-	1	-	-
Smoking	0.239	7.000	0.036–2.297	0.416	0.467	0.075–2.923	0.381	0.410	0.056–3.014
Time: Trauma-Surgery	0.808	0.952	0.642–1.413	0.064	0.656	0.421–1.024	0.385	1.195	0.799–1.787
Age	0.697	0.989	0.934–1.047	0.581	0.987	0.942–1.034	0.924	0.998	0.956–1.042
Sex	1.000	1.000	0.141–7.099	0.820	1.200	0.250–5.768	0.156	0.303	0.058–1.576

**Table 6:** Effect of risk factors on outcome of split repair showing the significance level *p*, the hazard ratio HR, and the confidence interval CI.

Split-Repair		ATM			EHL			PM	
	*p*	HR	CI	*p*	HR	CI	*p*	HR	CI
Obesity	0.447	1.122	0.834–1.151	0.676	0.935	0.684–1.280	0.622	0.904	0.607–1.348
Arterial Hypertension	1	-	-	1	-	-	1	-	-
Diabetes mellitus	0.530	1.500	-	0.372	0.578	-	0.08	4.000	-
Smoking	0.530	1.500	-	1	-	-	0.08	4.000	-
Time: Trauma-Surgery	0.796	1.118	0.481–2.597	0.657	0.852	0.419–1.729	0.998	-	-
Age	0.106	1.089	0.982–1.207	0.179	0.945	0.871–1.026	0.981	1.004	0.923–1.093
Sex	0.779	1.500	0.089–25.392	0.482	2.500	0.194–32.194	0.748	0.600	0.027–13.582
Distance > 8 cm	0.530	1.500	-	0.372	0.578	-	0.08	4.00	-

**Table 7:** Effect of risk factors on outcome of autologous nerve grafting showing the significance level *p*, the hazard ratio HR, and the confidence interval CI.

Nerve Grafting		ATM			EHL			PM	
	*p*	HR	CI	*p*	HR	CI	*p*	HR	CI
Obesity	0.926	0.926	0.858–1.150	0.364	0.920	0.768–1.102	0.138	1.142	0.958–1.362
Arterial Hypertension	0.826	0.750	0.058–9.719	1	1	-	1	-	-
Diabetes mellitus	1	-	-	1	-	-	0.678	1.857	-
Smoking	0.531	0.458	0.040–5.256	1	1	0.075–13.367	0.267	4.333	0.326–57.649
Time: Trauma-Surgery	0.943	1.012	0.735–1.393	0.398	0.854	0.591–1.233	0.450	1.140	0.812–1.602
Age	0.943	1.012	0.735–1.393	0.222	0.959	0.897–1.025	0.413	0.974	0.913–1.038
Sex	0.291	3.000	0.390–23.072	0.164	4.500	0.542–37.378	0.848	1.2223	0.158–9.467
Distance > 8 cm	0.203	0.320	0.055–1.847	0.037	0.109	0.14–0.872	0.514	0.556	0.095–3.246

**Table 8:** Effect of different stages of obesity on functional outcome of the entire patient collective showing the significance level *p*, the hazard ratio HR, and the confidence interval CI.

Obesity		ATM	
	*p*	HR	CI
Obesity Grade I	0.380	0.629	0.224–1.769
Obesity Grade II	0.250	2.625	0.507–13.591
Obesity Grade III	0.999	-	-

**Table 9:** Sensory deficit and pain before surgery and more than 1 year after surgery.

	Sensory Deficit		Pain	
	Before Surgery	More than 1 Year after Surgery	Before Surgery	More than 1 Year after Surgery
Neurolysis	33	19	10	5
Split-Repair	14	9	3	5
Nerve graft	33	30	7	8

## Data Availability

The data are available upon request.
